# Hepatobiliary surgery based on intelligent image segmentation technology

**DOI:** 10.1515/biol-2022-0674

**Published:** 2023-08-30

**Authors:** Fuchuan Wang, Chaohui Xiao, Tianye Jia, Liru Pan, Fengxia Du, Zhaohai Wang

**Affiliations:** Faculty of Hepatology Medicine, Chinese People’s Liberation Army (PLA) General Hospital, Beijing 100039, China; Faculty of Hepato-Biliary-Pancreatic Surgery, Chinese People’s Liberation Army (PLA) General Hospital, Beijing 100853, China; Department of Laboratory, Fifth Medical Center, Chinese People’s Liberation Army (PLA) General Hospital, Beijing 100039, China

**Keywords:** hepatological surgery, image segmentation technology, artificial intelligence, postoperative adverse reactions, intelligent optimization algorithm

## Abstract

Liver disease is an important disease that seriously threatens human health. It accounts for the highest proportion in various malignant tumors, and its incidence rate and mortality are on the rise, seriously affecting human health. Modern imaging has developed rapidly, but the application of image segmentation in liver tumor surgery is still rare. The application of image processing technology represented by artificial intelligence (AI) in surgery can greatly improve the efficiency of surgery, reduce surgical complications, and reduce the cost of surgery. Hepatocellular carcinoma is the most common malignant tumor in the world, and its mortality is second only to lung cancer. The resection rate of liver cancer surgery is high, and it is a multidisciplinary surgery, so it is necessary to explore the possibility of effective switching between different disciplines. Resection of hepatobiliary and pancreatic tumors is one of the most challenging and lethal surgical procedures. The operation requires a high level of doctors’ experience and understanding of anatomical structures. The surgical segmentation is slow and there may be obvious complications. Therefore, the surgical system needs to make full use of the relevant functions of AI technology and computer vision analysis software, and combine the processing strategy based on image processing algorithm and computer vision analysis model. Intelligent optimization algorithm, also known as modern heuristic algorithm, is an algorithm with global optimization performance, strong universality, and suitable for parallel processing. This algorithm generally has a strict theoretical basis, rather than relying solely on expert experience. In theory, the optimal solution or approximate optimal solution can be found in a certain time. This work studies the hepatobiliary surgery through intelligent image segmentation technology, and analyzes them through intelligent optimization algorithm. The research results showed that when other conditions were the same, there were three patients who had adverse reactions in hepatobiliary surgery through intelligent image segmentation technology, accounting for 10%. The number of patients with adverse reactions in hepatobiliary surgery by conventional methods was nine, accounting for 30%, which was significantly higher than the former, indicating a positive relationship between intelligent image segmentation technology and hepatobiliary surgery.

## 1 Introduction

With the development of science and technology, and the continuous improvement in medical image processing technology, how to classify various surgical procedures has become an important problem faced by surgeons. The most complex type of operation is hepatobiliary surgery, therefore, surgeons need to have rich clinical experience to be competent to block various complex organs in surgery. The operation requires the doctor to have a strong preoperative inspection ability, and to accurately locate the operation site, so as to accurately segment organs. In this process, there may be subtle differences between organs in different regions, so the division of each organ is very important.

Preoperative examination requires multiple manual operations to ensure clear images. In the process of operation, due to the lack of signal transmission between organ regions, doctors are often unable to quickly determine important organs and accurately grasp the surgical boundary, thus affecting the surgical effect. In addition, it may cause injury to patients due to incorrect operation. Therefore, it is of great significance to complete the operation efficiently and patient rehabilitation accurately and in a timely manner. In this process, image segmentation is one of the surgical steps, so it must be well and effectively applied to reduce surgical errors. Intelligent image segmentation technology has been applied in the field of intelligent medicine, especially in the medical field.

Based on this, this work studies hepatobiliary surgery based on intelligent image segmentation technology, and uses scientific methods to analyze it, to verify the relationship between the two. The innovation of this work was to combine intelligent image segmentation technology with liver surgery, which is not only a new attempt, but also in line with the development trend of modern medicine. This study not only has a novel perspective but also lays a foundation for liver surgery based on image segmentation technology, and provides new ideas and means for the treatment of liver tumors.

## 2 Related work

With the development of clinical work, the improvement in the diagnostic level of hepatobiliary diseases has laid a good foundation for hepatectomy technology and achieved great results in surgical operations. Wang et al’s. research provided reference for the application of indocyanine green fluorescence technology in hepatobiliary surgery [1]. Tang et al. found that in hepatobiliary surgery, augmented reality (AR) technology can help surgeons visualize the intrahepatic structure, so as to accurately operate and improve clinical results [2]. Mahdy et al’s. research showed that the infusion of terlipressin during hepatobiliary surgery can improve the portal vein hemodynamics during surgery, thereby reducing blood loss [3]. However, the above research works were carried out through the analysis of indocyanine green fluorescence technology and AR technology, lacking the research on image segmentation technology.

In view of the above problems, using intelligent image segmentation technology to analyze hepatobiliary surgery has become the research object of more and more scholars. Perica and Sun showed that the 3D printed liver model showed the liver anatomy and tumor with high accuracy, which can help preoperative planning and can be used to simulate the surgical process of treating malignant liver tumors [4]. Li et al. proposed a new semi supervised method for medical image segmentation and used it for liver segmentation of computed tomography (CT) scanning of liver tumor segmentation challenge dataset [5]. All these studies illustrate the applicability of image technology in the medical field, and provide rationality for the application of intelligent image segmentation technology to hepatobiliary surgery.

## 3 Construction of intelligent image segmentation technology

### 3.1 Overview of intelligent image segmentation technology

Intelligent image segmentation technology is an imaging technology that uses computer vision technology to extract, analyze, classify, retrieve, and mark images. With the development of artificial intelligence (AI) technology, AI has been widely used in the medical field, and image segmentation technology has broad application prospects in many fields. Computer vision in vision technology refers to the application of computer vision in various image processing fields, so as to realize the automatic recognition of various objects in images. At present, the most widely used and mature vision segmentation method in clinical application is based on regular neural network and machine learning algorithm, which can precisely locate the organs and tissues around the tumor. However, for liver tumors, the most widely used method is image segmentation algorithm based on depth learning. Intelligent image segmentation technology combines the advantages of machine learning method and deep learning technology, making it fast, accurate, less delay, interpretable, safe, and so on.

#### 3.1.1 Introduction to image segmentation

**Figure 1:**
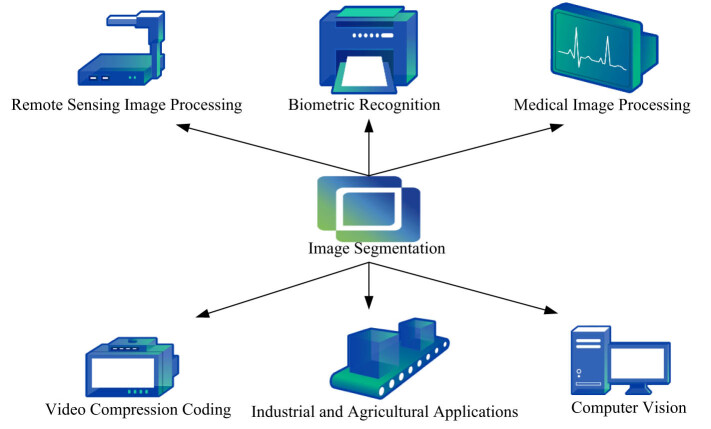
Application of image segmentation.

Image segmentation is the core of the whole image processing, which is directly related to the subsequent processing. Image segmentation is the technology and process of dividing an image into several specific regions with unique properties and proposing interested objects. It is a key step from image processing to image analysis [6,7]. Image segmentation can be defined as dividing an image into similar areas that do not overlap each other. The area here can be regarded as a connected set of pixels, that is, a set of pixels where all pixels are adjacent or in contact. Image segmentation has been widely used in various fields, as shown in Figure 1.

#### 3.1.2 Medical image segmentation technology

Medical image segmentation can be divided into three categories: manual segmentation, semi-automatic segmentation, and fully automatic segmentation. Manual segmentation mainly depends on the experience of doctors. In large-scale slice sequences, this method has high accuracy, but it requires a lot of work and time. The semi-automatic segmentation is realized by using the man–machine interface [8]. Automatic image segmentation is mainly realized by using fuzzy theory, neural network, and other related technologies.

**Figure 2:**
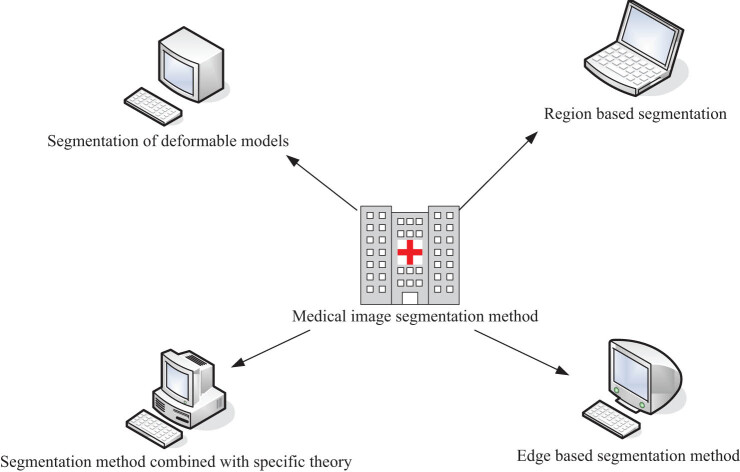
Method division of medical image segmentation.

The methods of medical image segmentation are divided into different categories, as shown in Figure 2.

The main applications of medical image segmentation are as follows: As the basis of image 3D reconstruction, 3D reconstruction algorithms or reconstruction software are used to achieve the reconstruction of three-dimensional structures. The statistics of characteristic parameters can be conducted to guide clinical diagnosis. Image compression is available for easy storage and management. The imaging mechanism of medical images is very complex, and its characteristics are fuzziness and non-uniformity. For example, the anatomy of the liver is similar to the gray scale of the surrounding tissues. When it is separated, there would be great adhesion, and it is difficult to separate the liver from the background [9,10]. Therefore, this study must recognize the technology and methods of medical image segmentation, use intelligent optimization algorithm to analyze the threshold of image segmentation technology, and segment the liver and gallbladder from the background [11,12].

#### 3.1.3 Principle and method of intelligent segmentation technology

Computer vision is a science that studies how to make a machine “see.” Furthermore, it refers to the use of cameras and computers to replace human eyes for machine vision such as target recognition, tracking, and measurement, and further graphic processing to make computer processing more suitable for human eyes to observe or transmit images to instruments for detection [13]. The intelligent segmentation system is composed of image acquisition system, computer vision analysis software, and computer vision analysis model. CT means computerized tomography. It uses accurately collimated X-ray beam, γ ray, ultrasonic wave, etc., to scan one section after another around a certain part of the human body together with a highly sensitive detector. It has the characteristics of fast scanning time, clear images, etc., and can be used for the inspection of various diseases [14]. It can be divided into X-ray CT (X-CT) and γ ray CT (γ-CT) according to different rays used. First, CT images of patients were collected, including head plain scan and whole-body plain scan, and the lesions were diagnosed, classified, and resected by image processing algorithms and computer vision analysis model processing methods. The computer vision analysis model uses machine learning algorithm to extract image features and train the model. Image segmentation includes edge detection and feature extraction. The purpose of edge detection is to determine the edge and contour of the image (such as lesions and boundaries) [15,16]. Feature extraction can extract tumor edges and features at different scales (including edge segmentation) from the sample set. In the algorithm design, both processing space and image processing algorithm shall be taken into account to process local features, which can effectively improve the information required for segmentation of liver tumor tissue [17,18]. CT images can be used to perform cluster analysis from the perspective of the patient’s body to predict the patient’s focus type and tumor morphology, and then different features can be manually selected for further segmentation [19,20]. For example, when it is determined that the liver has a tumor, the ratio of tumor volume to tumor length can be used to judge the prognosis. When the tumor is located in the left lobe of the liver, intelligent segmentation is required. The tumor growing in the central area of the liver needs to be accurately segmented. However, there are some problems such as low segmentation accuracy, unsatisfactory segmentation, and possible errors when segmenting the whole. During prediction, the predicted image shall be segmented after being detected. In addition, due to the special location of hepatic portal vein, classification and extraction are required to ensure the accuracy and efficiency of clinical surgery. The combination of AI technology and computer vision analysis software designed to improve classification accuracy and save operation time can greatly improve its efficiency and accuracy.

### 3.2 Application of intelligent optimization algorithm in image threshold segmentation

#### 3.2.1 Basic concepts of intelligent optimization algorithm

Intelligent optimization algorithm is a calculation method based on the social behavior of biological groups or the laws of natural phenomena. It is a random search algorithm based on bionics (biological intelligence) and simulation (physical phenomena). The optimal problem is a mathematical problem that finds the optimal solution from a large number of candidate solutions under the condition of meeting specific constraints. Mechanical design is to conceive, analyze, and calculate the working principle, structure, motion mode, transmission mode of force and energy, material, shape and size of each part, lubrication method, etc., of the machine according to the use requirements, and convert them into specific descriptions as the working process of manufacturing basis. Optimization problems generally exist in many fields of scientific research, such as enterprise production, signal processing, image processing, social management, automatic control, and mechanical design. At present, many optimization methods have been widely used in many aspects and have achieved significant economic and social benefits. The optimization method optimizes the problem-solving efficiency, and cost and resource allocation, and the larger the optimization problem is, the more obvious its impact would be.

Engineering technology refers to practical engineering technology, also known as production technology, which is actually applied in industrial production. With the development of theory and engineering technology, many complex optimization problems are characterized by large-scale, nonlinear, high complexity, and multi extremum. In addition, many problems require real-time computing, which makes it difficult for traditional optimization algorithms to meet the actual needs. In order to solve this problem, finding an effective optimal solution has become an important topic in various fields.

Whether it is bionic or imitative, it reflects the concept of “learning from nature.” This optimal method is called “natural operation.”

#### 3.2.2 Basic principle of threshold segmentation

Threshold, also called critical value, refers to the lowest or highest value that an effect can produce. The basic idea of image threshold segmentation technology is to set a feature threshold according to certain standards, and divide each pixel in the image into corresponding regions. There are similar characteristics in each region, while there are significant differences between each region.

Assume that the size of the original image is \[N\times M]\] and the gray scale sequence is \[N]\] . Then, the pixel gray value with \[(a,b)]\] coordinate can be represented by the two-dimensional function \[O(a,b)]\] , here \[a\in {[}1,N]]\] , \[b\in {[}1,M]]\] , \[0\le O(a,b)\le Z-1]\] . Single threshold segmentation refers to dividing the whole image into two regions through the given feature threshold \[U]\] and expressing them as follows:

(1) \[H(a,b)=\left\{\begin{array}{c}{y}_{0},O(a,b)\lt U\\ {y}_{1},O(a,b)\ge U.\end{array}\right.]\]

In a gray image, the gray value of each pixel is compared with the set threshold value. The result shows that when the gray value is higher than the threshold value, the pixel is white, and when the gray value is lower than the threshold value, the pixel is black.

The single threshold segmentation method is used to segment a single object. If there are multiple objects and these objects are in different gray levels, the multi threshold method is used to separate them. Assuming that the selected threshold is \[m]\] , the segmented image can be expressed as follows:

(2) \[H(a,b)=\left\{\begin{array}{c}{z}_{0},0\lt O(a,b)\le {u}_{1}\\ {z}_{1},{u}_{1}\lt O(a,b)\le {u}_{2}\\ \vdots ,\hspace{1em}\vdots \\ {z}_{m},{u}_{m}\lt O(a,b)\le Z-1.\end{array}\right.]\]

Among them, \[{z}_{0},{z}_{1},\ldots ,{z}_{m}]\] and \[{u}_{0},{u}_{1},\ldots ,{u}_{m}]\] represent the different regions generated after image segmentation and the selected segmentation threshold, respectively.

#### 3.2.3 Maximum entropy method

If the gray level of an image is between \[{[}0,\hspace{.25em}Z-1]]\] , the possibility of having \[o]\] pixels in the image is \[{q}_{o}]\] . Then, the entropy of the image can be expressed using formula (3).

(3) \[J=-\mathop{\sum }\limits_{o=1}^{Z-1}({q}_{o})\mathrm{lg}({q}_{o}).]\]

The maximum entropy method is the most widely used threshold segmentation method at present. It assumes that the target area and the background area obey different probability distributions, and determines the segmentation threshold according to the maximum entropy. In the one-dimensional histogram, it is assumed that the \[u]\] threshold is used to segment the image, and the entropy values of the target and background area are \[{J}_{0}]\] and \[{J}_{1}]\] , respectively

(4) \[{J}_{0}=-\mathop{\sum }\limits_{o=1}^{u-1}\left(\frac{{q}_{o}}{{e}_{0}}\right)\mathrm{lg}\left(\frac{{q}_{o}}{{e}_{0}}\right),]\]

(5) \[{J}_{1}=-\mathop{\sum }\limits_{o=1}^{Z-1}\left(\frac{{q}_{o}}{{e}_{1}}\right)\mathrm{lg}\left(\frac{{q}_{o}}{{e}_{1}}\right).]\]

In this case, the entropy of the whole image is as follows:

(6) \[J={J}_{0}+{J}_{1}.]\]

The best segmentation threshold \[{u}^{\ast }]\] selected should conform to the following:

(7) \[{u}^{\ast }=\mathop{\text{arg}\hspace{.25em}\max }\limits_{0\le u\le Z-1}\{J(u)\}.]\]

### 3.3 General flow of intelligent optimization algorithm for image threshold segmentation

**Figure 3:**
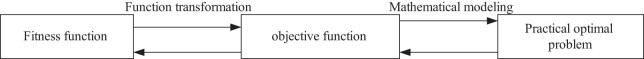
The flow of intelligent optimization algorithm in solving practical optimal problems.

When solving the practical optimal problem, the intelligent optimization algorithm should first establish an accurate mathematical model and determine the objective function of the problem to be optimized. Second, the objective function is transformed into the adaptive function in the intelligent optimization algorithm. Finally, intelligent optimization methods can be used to find the optimal adaptability and corresponding optimal solution, and apply them to the actual optimization problems, as shown in Figure 3.

## 4 Experimental study on hepatobiliary surgery based on intelligent image segmentation technology

### 4.1 Preparation before experiment

#### 4.1.1 Determination of experimental objects

From January 2021 to April 2021, a total of 60 patients undergoing hepatobiliary surgery were studied experimentally.

Inclusion criteria are as follows: male and female, age 50–75, American Society of Anesthesiologists (ASA) grade I or II, height 160–175 cm, weight 55–65 kg, no other diseases. ASA is a physician association organization engaged in education, research, and scientific research, aiming to improve and maintain the anesthesia related standards in the medical industry and improve the monitoring of patients.

Hepatocellular carcinoma (HCC): It is the most common type of carcinoma. HCC can be divided into primary and secondary types clinically, of which primary HCC is the most common malignant tumor of the liver. The liver is located in the front of the abdomen of the human body, which is spherical or oval in shape. Its epithelial cells would spread around after being damaged, so the mortality of liver cancer is very high.


**Informed consent:** Informed consent has been obtained from all individuals included in this study.
**Ethical approval:** The research related to human use has been complied with all the relevant national regulations, institutional policies and in accordance with the tenets of the Helsinki Declaration, and has been approved by the authors' institutional review board or equivalent committee.

#### 4.1.2 Grouping of subjects

In this study, 60 patients were divided into 2 groups by stratified random method, 30 patients in each group. The experiment was divided into experimental group and control group. The hepatobiliary surgery was performed on the experimental group based on intelligent image segmentation technology (Group X), while the same was performed on the control group based on conventional methods (Group Y).

#### 4.1.3 Experimental observation index

This study made detailed statistics on age, sex, weight, operation time, stay time in the anesthesia recovery room, first time out of bed after operation, length of hospital stay, occurrence and number of adverse events after operation, postoperative pain score, patient satisfaction, etc.

#### 4.1.4 Experimental scoring standard

##### 4.1.4.1 Visual analysis scale (VAS) scoring standard

All patients were followed up according to the corresponding time points, and the pain situation of 60 patients during the rehabilitation process was counted, and VAS score was taken as the standard. The VAS score is as follows:

0 point: no pain; 1–3 points: mild pain, which would not affect sleep; 4–6 points: moderate pain, which affects sleep and tolerable; 7–10: severe pain, unbearable, and would seriously affect appetite and sleep.

##### 4.1.4.2 Patient satisfaction evaluation

The score is divided into five grades, A–E, of which A is very dissatisfied; B is not satisfied; C is satisfied; D is quite satisfied; E is extremely satisfied.

#### 4.1.5 Experimental statistical treatment

In this study, Statistical Product Service Solutions and GraphPad Prism are used for statistical analysis. All tests are bilateral. The size of *P* value is used to determine whether it is statistically significant, that is, whether *P* value is less than 0.05.

### 4.2 Experimental results

#### 4.2.1 General information of the patient

The age, weight, operation time, and sex of the two groups of patients are compared, and the results are shown in Table 1.

**Table 1:** Comparison of general conditions of two groups of patients

Index	Grouping		*P* value
	*X*	*Y*	
Age	65.30 ± 8.16	65.50 ± 7.65	0.842
Weight (kg)	60.70 ± 12.1	61.20 ± 11.32	0.718
Operation time (min)	200 ± 20.54	230 ± 18.68	0.979
Gender (male/female)	16/14	15/15	0.944

Table 1 shows that the *P* values of the two groups in terms of age, weight, operation time, and gender are 0.842, 0.718, 0.979, and 0.944, respectively. *P* values were significantly greater than 0.05, indicating that there was no statistically significant difference between the two groups of patients, which was comparable.

#### 4.2.2 Postoperative condition of the patient

**Figure 4:**
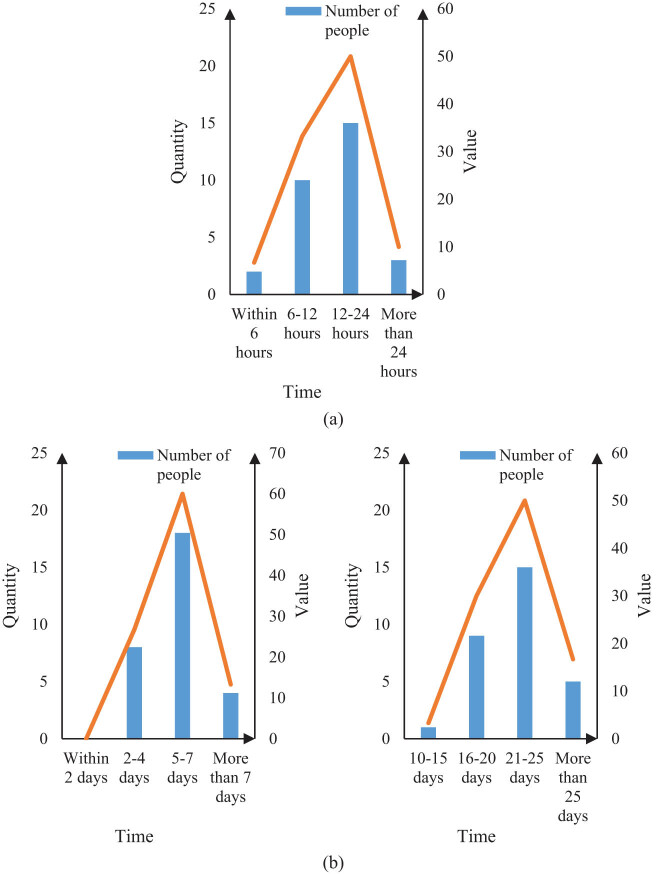
Time comparison of patients in group Y under three conditions. (a) Stay time in the anesthesia recovery room of group Y. (b) First time out of bed after operation in group Y, and (c) hospitalization days in group Y.

**Figure 5:**
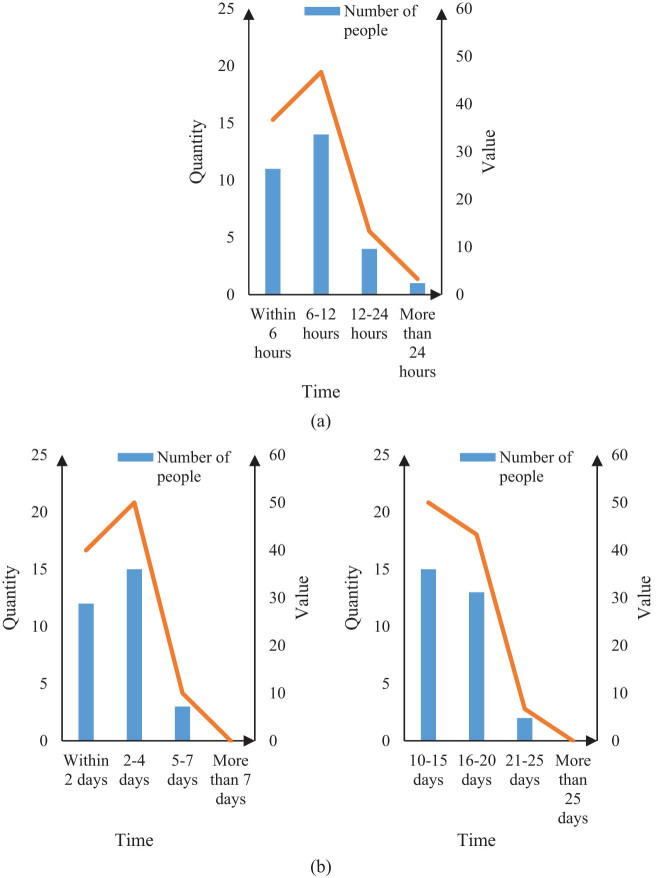
Time comparison of patients in Group X under three conditions. (a) Time of stay in the anesthesia recovery room of group X. (b) Time of getting out of bed for the first time after operation in group X and (c) hospitalization days in group X.

The time of stay in the anesthesia recovery room, the time of first getting out of bed after surgery, and the number of days in the hospital are compared between the two groups of patients. The results are shown in Figures 4 and 5.

It can be seen from Figure 4(a) that 2 patients in Group Y stayed in the anesthesia recovery room for 6 h, accounting for 6.7%; 10 patients stayed for 6–12 h, accounting for 33.3%. There are 15 people from 12 to 24 h, accounting for 50%, and 3 people for more than 24 h, accounting for 10%. It can be seen from Figure 4(b) that the number of patients in Group Y who first got out of bed within 2 days after surgery is 0, and the number of patients in 2–4 days is 8, accounting for 26.7%. There are 18 people in 5–7 days, accounting for 60%, and 4 people in more than 7 days, accounting for 13.3%. It can be seen from Figure 4(c) that the number of patients in Group Y with hospitalization days of 10–15 days is 1, accounting for 3.3%, and the number of patients in 16–20 days is 9, accounting for 30%. The number of people in 21 to 25 days is 15, accounting for 50%, and the number of people in more than 25 days is 5, accounting for 16.7%. It can be seen from Figure 4 that most patients undergoing routine hepatobiliary surgery recover from anesthesia within 6–24 h, get out of bed for the first time within 2–7 days, and stay in hospital for 16–25 days.

It can be seen from Figure 5(a) that 11 patients in Group X stayed in the anesthesia recovery room for 6 h, accounting for 36.7%, and 14 patients stayed for 6–12 h, accounting for 46.7%. There were 4 persons in 12–24 h, accounting for 13.3%, and 1 person for more than 24 h, accounting for 3.3%. It can be seen from Figure 5(b) that the number of patients in Group X who first got out of bed within 2 days after surgery is 12, accounting for 40%. The number of people in 2–4 days is 15, accounting for 50%. The number of people in 5–7 days is 3, accounting for 10%, and there were no patients in the more than 7 days category. It can be seen from Figure 5(c) that the number of patients in Group X with hospitalization days of 10–15 days is 15, accounting for 50%, and the number of patients in 16–20 days is 13, accounting for 43.3%. There are 2 people from 21 to 25 days, accounting for 6.7%, and nobody in the more than 25 days group. It can be seen from Figure 5 that most patients undergoing hepatobiliary surgery based on intelligent image segmentation technology recovered from anesthesia within 12 h, got out of bed for the first time within 4 days, and stayed in hospital for 10–20 days.

To sum up, the hepatobiliary surgery based on intelligent image segmentation technology is obviously superior to the conventional hepatobiliary surgery for the duration of the patient’s stay in the anesthesia recovery room, the first time to get out of bed after surgery, and the number of days in hospital.

#### 4.2.3 Comparison of VAS scores between two groups

**Figure 6:**
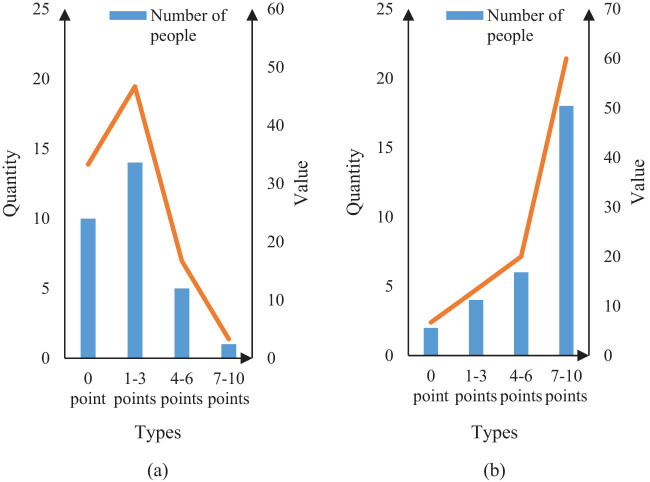
VAS pain scores of patients in both groups at rest. (a) Pain of patients in group X and (b) pain of patients in group Y.

Because the rest of the patient is also helpful for the recovery of the body, it is necessary to ensure that the patient has enough sleep after the operation. For this reason, this study analyzed the statistics on the rest of the two groups of patients after surgery, and obtained the results using VAS scores, as shown in Figure 6.

It can be seen from Figure 6(a) that 10 people in Group X scored 0 in VAS, accounting for 33.3%, and 14 people scored 1–3, accounting for 46.7%. Five people scored 4–6, accounting for 16.7%, and one person scored 7–10, accounting for 3.3%. It can be seen from Figure 6(b) that there are 2 persons in Group Y with VAS score of 0, accounting for 6.7%, and 4 persons with VAS score of 1–3, accounting for 13.3%. There are 6 persons with 4–6 points, accounting for 20%, and 18 persons with 7–10 points, accounting for 60%. It can be seen from Figure 6 that, according to the analysis of patients’ sleep quality, the number of patients with good sleep quality in Group X is significantly more than that in Group Y. It is more beneficial for patients’ health to perform hepatobiliary surgery with intelligent image segmentation technology than with conventional methods.

#### 4.2.4 Comparison of patient satisfaction evaluation between two groups after operation

**Figure 7:**
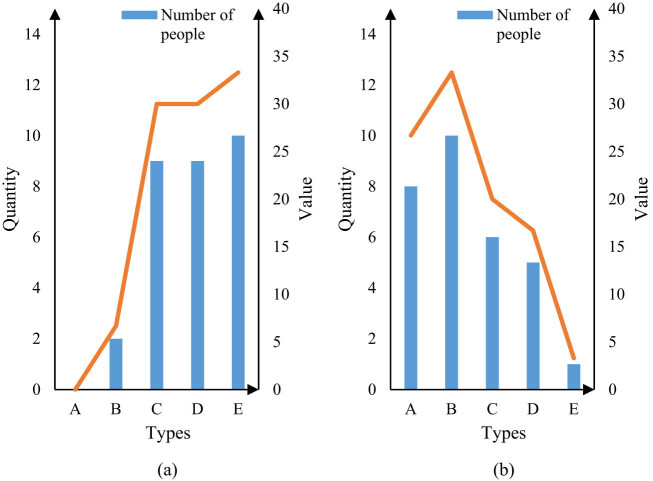
Satisfaction evaluation of two groups of patients. (a) Satisfaction evaluation of group X and (b) satisfaction evaluation of group Y.

The satisfaction of the two groups of patients with their respective surgical results was investigated and counted, as shown in Figure 7.

It can be seen from Figure 7(a) that there are two people in Group X who are dissatisfied with the surgical results, accounting for 6.7%, and nine people are both satisfied and quite satisfied, accounting for 30%. The most satisfied patients were 10, accounting for 33.3%. There were no very dissatisfied patients. It can be seen from Figure 7(b) that eight people in Group Y were very dissatisfied with the surgical results, accounting for 26.7%. ten people were dissatisfied, accounting for 33.3%; six people were relatively satisfied, accounting for 20%. Five people were satisfied, accounting for 16.7%, and one person was very satisfied, accounting for 3.3%. It can be seen from Figure 7 that the satisfaction evaluation of patients in Group X on the surgical results is very good, while the satisfaction evaluation of patients in Group Y on the surgical results is not satisfactory, which further shows that the intelligent image segmentation technology is helpful for hepatobiliary surgery.

#### 4.2.5 Comparison of adverse reactions after operation

**Figure 8:**
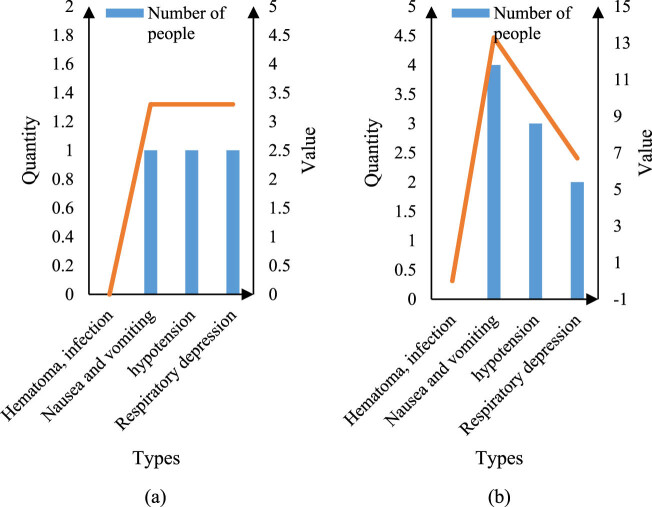
Postoperative adverse reactions. (a) Number of adverse reactions in group X and (b) number of adverse reactions in group Y.

In order to explain the relationship between intelligent image segmentation technology and hepatobiliary surgery more scientifically, this study analyzed the statistics on the adverse reactions and the number of patients after surgery. The results are shown in Figure 8.

It can be seen from Figure 8(a) that 1 case of nausea, vomiting, hypotension, and respiratory depression occurred in adverse reactions of Group X, accounting for 3.3%. It can be seen from Figure 8(b) that the number of nausea, vomiting, hypotension, and respiratory depression in adverse reactions of group Y was 4, 3, and 2, respectively, accounting for 13.3, 10, and 6.7%. It can be seen from Figure 8 that none of the patients had hematoma or infection, but other adverse reactions are obviously different. The number of adverse reactions in group X was three, accounting for 10%. The number of adverse reactions in group Y was nine, accounting for 30%. This shows that intelligent image segmentation technology can reduce the probability of bad reactions for patients after surgery.

## 5 Conclusion

The application of intelligent image segmentation technology is helpful to solve the practical problems in the accurate classification of liver tumors. At present, the doctors of liver surgery are highly demanding, the manual operation takes a long time, the patients are injured, and have many complications. The patients recovered slowly and had poor quality of life. AI image segmentation technology has the characteristics of high intelligence, which can quickly and effectively segment liver tissue accurately and effectively. It has high research value on the morphological characteristics and anatomical structure of liver cells. Intelligent CT has a very good development prospect. It can realize unattended operation, accumulate relevant knowledge and experience through learning, and solve practical problems through real-time surgery. In addition, image segmentation technology based on AI can effectively solve many difficult problems in the traditional surgery process, which plays a key role in the success of surgery. In other words, the application prospect of AI-based image segmentation technology in the diagnosis and treatment of liver diseases is broad, which can effectively improve the success rate of liver surgery and the therapeutic effect, and provide strong support and help for the diagnosis and treatment of liver and biliary surgery diseases in China. Due to the lack of comprehensive personal knowledge and ability, this work only studied 60 patients in a university affiliated hospital, and the number of experimental subjects was small. Although it is representative to some extent, it would still make the conclusion of the article questionable, which is not conducive to the development of intelligent image segmentation technology in liver surgery. It is hoped that more people and teams would join in this research in the future to expand the research objects and make the experimental conclusions more scientific.

## References

[1] Wang X, Teh CS, Ishizawa T, Aoki T, Cavallucci D, Lee SY, et al. Consensus guidelines for the use of fluorescence imaging in hepatobiliary surgery. Ann Surg. 2021;274(1):97–106. 10.1097/SLA.0000000000004718 33351457

[2] Tang R, Ma LF, Rong ZX, Li MD, Zeng JP, Wang XD, et al. Augmented reality technology for preoperative planning and intraoperative navigation during hepatobiliary surgery: a review of current methods. Hepatobiliary Pancreat Dis Int. 2018;17(2):101–12. 10.1016/j.hbpd.2018.02.002 29567047

[3] Mahdy MM, Abbas MS, Kamel EZ, Mostafa MF, Herdan R, Hassan SA, et al. Effects of terlipressin infusion during hepatobiliary surgery on systemic and splanchnic haemodynamics, renal function and blood loss: a double-blind, randomized clinical trial. BMC Anesthesiol. 2019;19(1):1–9. 10.1186/s12871-019-0779-6 PMC6570915 31200638

[4] Perica ER, Sun Z. A systematic review of three-dimensional printing in liver disease. J Digit Imaging. 2018;31(5):692–701. 10.1007/s10278-018-0067-x PMC6148823 29633052

[5] Li X, Yu L, Chen H, Fu CW, Xing L, Heng PA. Transformation-consistent self-ensembling model for semisupervised medical image segmentation. IEEE Trans Neural Netw Learn Syst. 2020;32(2):523–34. 10.1109/TNNLS.2020.2995319 32479407

[6] Estevao M. Design and implementation of intelligent fault diagnosis system for construction machinery supporting wireless communication network. Kinetic Mech Eng. 2020;1(3):17–24. 10.38007/KME.2020.010303.

[7] Kavita U. Visual intelligent recognition system based on visual thinking. Kinetic Mech Eng. 2021;2(1):46–54. 10.38007/KME.2021.020106.

[8] Shan PF. Image segmentation method based on K-mean algorithm. EURASIP J Image Video Process. 2018;2018:81. 10.1186/s13640-018-0322-6.

[9] Zhao X, Song P, Zhang Y, Huang J. Performing laparoscopic surgery – Perspectives of young Chinese hepatobiliary surgeons. Biosci Trends. 2018;12(2):208–10. 10.5582/bst.2018.01086 29760360

[10] Lee JH, Yoon CJ, Choi WS. Transhepatic stent placement for portal vein obstruction after hepatobiliary and pancreatic surgery: long-term efficacy and risk factor for stent failure. Eur Radiol. 2021;31(3):1300–7. 10.1007/s00330-020-07139-3 32880695

[11] Park CJ, Armenia SJ, Cowles RA. Trends in routine and complex hepatobiliary surgery among general and pediatric surgical residents: what is the next generation learning and is it enough? J Surg Educ. 2019;76(4):1005–14. 10.1016/j.jsurg.2019.02.007 30902561

[12] Chen-Xu J, Bessa-Melo R, Graça L, Costa-Maia J. Incisional hernia in hepatobiliary and pancreatic surgery: incidence and risk factors. Hernia. 2019;23(1):67–79. 10.1007/s10029-018-1847-4 30392165

[13] Du H, Wang J, Liu M, Wang Y, Meijering E. SwinPA-Net: Swin transformer based multiscale feature pyramid aggregation network for medical image segmentation. IEEE Trans Neural Netw Learn Syst. 2022;1–12. 10.1109/TNNLS.2022.3204090. 36121961

[14] Jiang Y, Chen W, Liu M, Wang Y. 3D neuron microscopy image segmentation via the ray-shooting model and a DC-BLSTM network. IEEE Trans Med Imaging. 2021;40(1):26–37. 10.1109/TMI.2020.3021493 32881683

[15] Yasuda J, Okamoto T, Onda S, Fujioka S, Yanaga K, Suzuki N, et al. Application of image‐guided navigation system for laparoscopic hepatobiliary surgery. Asian J Endosc Surg. 2020;13(1):39–45. 10.1111/ases.12696 30945434

[16] Saito Y, Sugimoto M, Morine Y, Imura S, Ikemoto T, Yamada S, et al. Intraoperative support with three-dimensional holographic cholangiography in hepatobiliary surgery. Langenbeck’s Arch Surg. 2022;407(3):1285–9. 10.1007/s00423-021-02336-0 34557939

[17] Lillemoe HA, Aloia TA. Enhanced recovery after surgery: hepatobiliary. Surg Clin. 2018;98(6):1251–64. 10.1016/j.suc.2018.07.011 PMC6345553 30390857

[18] Krautz C, Gall C, Gefeller O, Nimptsch U, Mansky T, Brunner M, et al. In-hospital mortality and failure to rescue following hepatobiliary surgery in Germany-a nationwide analysis. BMC Surg. 2020;20(1):1–11. 10.1186/s12893-020-00817-5 PMC7388497 32727457

[19] Lambert Joel E, Hayes Lawrence D, Keegan Thomas J, Subar Daren A, Gaffney Christopher J. The impact of prehabilitation on patient outcomes in hepatobiliary, colorectal, and upper gastrointestinal cancer surgery: a PRISMA-accordant meta-analysis. Ann Surg. 2021;274(1):70–7. 10.1097/SLA.0000000000004527 33201129

[20] Zhang XP, Gao YZ, Chen ZH, Chen MS, Li LQ, Wen TF, et al. An eastern hepatobiliary surgery hospital/portal vein tumor thrombus scoring system as an aid to decision making on hepatectomy for hepatocellular carcinoma patients with portal vein tumor thrombus: a multicenter study. Hepatology. 2019;69(5):2076–90. 10.1002/hep.30490 30586158

